# Net energy of corn, soybean meal and rapeseed meal in growing pigs

**DOI:** 10.1186/s40104-017-0169-1

**Published:** 2017-05-01

**Authors:** Zhongchao Li, Yakui Li, Zhiqian Lv, Hu Liu, Jinbiao Zhao, Jean Noblet, Fenglai Wang, Changhua Lai, Defa Li

**Affiliations:** 1 State Key Laboratory of Animal Nutrition, Ministry of Agriculture Feed Industry Centre, China Agricultural University, No. 2, Yuanminyuan west road, Haidian district, Beijing, 100193 China; 2 INRA, UMR Pegase, 35590 Saint-Gilles, France

**Keywords:** Caloric efficiency, Growing pig, Heat production, Net energy, Rapeseed meal

## Abstract

**Background:**

Two experiments were conducted to estimate the net energy (NE) of corn, soybean meal, expeller-pressed rapeseed meal (EP-RSM) and solvent-extracted rapeseed meal (SE-RSM) using indirect calorimetry and to validate the NE of these four ingredients using pig growth performance.

**Methods:**

In Exp.1, 24 barrows (initial BW = 36.4 ± 1.6 kg) were allotted to 1 of 4 diets which included a corn basal diet, a corn-soybean meal basal diet and two rapeseed meal diets containing 20% EP-RSM (9.5% ether extract) or SE-RSM (1.1% ether extract) substituted for corn and soybean meal. The design allowed the calculation of NE values of corn, soybean meal and rapeseed meals according to the difference method. In Exp.2, 175 growing pigs (initial BW = 36.0 ± 5.2 kg) were fed 1 of 5 diets for 28 d, with five pigs per pen and seven replications (pens) per treatment in order to validate the measured energy values. Diets were a corn-soybean meal diet and four diets including 10% or 20% EP-RSM and 10% or 20% SE-RSM.

**Results:**

The NE of corn, soybean meal, EP-RSM and SE-RSM were 12.46, 11.34, 11.71 and 8.83 MJ/kg DM, respectively. The NE to ME ratio of corn (78%) was similar to tabular values, however, the NE to ME ratios of soybean meal (70%) and rapeseed meal (76%) were greater than tabular values. The greater NE value in EP-RSM than in SE-RSM is consistent with its higher EE content. Increasing EP-RSM or SE-RSM did not affect the growth performance of pigs and the caloric efficiency of NE was comparable for all diets.

**Conclusions:**

The NE of EP-RSM was similar to soybean meal, and both were greater than SE-RSM. The DE, ME and NE values measured in Exp.1 are confirmed by results of Exp. 2 with comparable caloric efficiencies of DE, ME or NE for all diets.

## Background

Following soybean, the most abundant oilseed produced in the world is rapeseed, with a production of about 70 million tons in 2015 [1]. There are two primary rapeseed co-products associated with oil extraction processing methods: expeller-pressed rapeseed meal (EP-RSM) containing 10% to 15% residual oil and solvent-extraction rapeseed meal (SE-RSM) containing much less oil (1–2%) than EP-RSM. In a previous work, the DE, ME and standardized ileal digestible (SID) amino acid (AA) of SE-RSM [2] and EP-RSM [3] were evaluated. Little et al. [4] found high-protein or conventional rapeseed meal may fully replace soybean meal as protein supplements in growing-finishing pig diets without impairing pig performance or carcass quality. However, there are limited reports that estimate and compare the NE of EP-RSM and SE-RSM or that have validated the NE value of rapeseed meals using a growth trial. In addition, there is no clear agreement on the proper method of validation of energy values using growth trials. Some researchers have proposed the concept of caloric efficiency [5–8] meaning that, if the assigned energy value is correct, regardless of the test ingredient inclusion level, a similar caloric efficiency (ie, dietary energy per kg of BW gain) will be calculated among the dietary treatments. However, in these experiments, the NE value of corn and soybean meal, which are the two main ingredients in the formula, were referenced from tabular values. However, in the current experiment, we have measured the NE of all ingredients in a first experiment (Exp. 1) and used the obtained values in a second validation experiment (Exp. 2). Practically, the NE of corn, soybean meal, EP-RSM and SE-RSM were determined by indirect calorimetry and these four ingredients were used subsequently to prepare five diets fed in a growth trial.

## Methods

The two experimental protocols used in this study were approved by the Institutional Animal Care and Use Committee of China Agricultural University (Beijing, China).

### Exp.1: Net energy experiment

Twenty-four barrows (Duroc × Large White × Landrace, initial BW = 36.4 ± 1.6 kg) were used in this experiment conducted at the FengNing Swine Research Unit of China Agricultural University (Hebei Province, China). The 24 barrows were allotted to 1 of 4 dietary treatments with six pigs per diet and four periods. Diets included a corn basal diet supplemented with free amino acids in order to improve the amino acids balance of corn and maximize protein gain, a corn-soybean meal basal diet (to calculate the energy of soybean meal using the difference method), and two rapeseed meal diets containing 20% EP-RSM or SE-RSM substituted for corn and soybean meal (to calculate the energy of rapeseed meals using the difference method). The analyzed nutrient composition of ingredients are shown in Table 1. The ratios between SID essential amino acids and SID Lys met or exceeded the recommended amino acid ratios (NRC 2012) in all diets; but the amino acids content in the corn diet was below the standard recommendations for growing pigs (Table 2).

**Table 1:** Analyzed nutrient composition of ingredients used in the experiment (%, DM basis)^a^

Item	Corn	SBM	EP-RSM	SE-RSM
GE, MJ/kg	18.44	19.50	21.33	19.54
Crude protein	9.2	48.5	39.2	42.4
Ether extract	3.0	1.2	9.5	1.1
NDF	14.0	17.1	37.0	30.8
ADF	2.5	10.0	22.8	20.7
Ash	1.2	6.2	6.4	7.0
Indispensable AA				
Arg	0.39	3.81	2.75	2.64
His	0.24	1.46	1.27	1.27
Leu	1.13	3.82	2.84	2.93
Ile	0.27	2.14	1.50	1.59
Lys	0.28	3.23	2.70	2.57
Met	0.16	0.57	0.75	0.77
Phe	0.41	2.46	1.52	1.41
Thr	0.36	2.12	1.77	1.89
Trp	0.06	0.61	0.61	0.65
Val	0.42	2.34	2.03	2.14
Dispensable AA				
Ala	0.65	2.25	1.91	1.98
Asp	0.62	5.74	2.82	2.97
Cys	0.17	0.63	0.95	0.95
Glu	1.69	8.77	7.18	7.31
Gly	0.32	2.21	2.14	2.22
Pro	0.86	2.72	2.86	2.92
Ser	0.45	2.54	1.72	1.83
Tyr	0.31	1.66	1.02	0.93

^a^All samples were analyzed in duplicate, *SBM* soybean meal, *EP-RSM* expelled press rapeseed meal, *SE-RSM* solvent extracted rapeseed meal

**Table 2:** Ingredients and chemical composition of the Exp.1 diets^a^

Diet	Corn	SBM	EP-RSM	SE-RSM
Ingredients, %				
Corn	97.03	77.13	61.31	61.31
Soybean meal	-	20.00	15.90	15.90
EP-RSM	-	-	20.00	0.00
SE-RSM	-	-	-	20.00
Dicalcium phosphate	0.90	0.90	0.90	0.90
Limestone	0.75	0.75	0.75	0.75
Salt	0.35	0.35	0.35	0.35
Vitamins and minerals premix^b^	0.50	0.50	0.50	0.50
Lys-HCl	0.26	0.21	0.16	0.16
DL-Met	0.14	0.11	0.09	0.09
L-Thr	0.03	0.02	0.02	0.02
L-Trp	0.04	0.03	0.03	0.03
DM, %	86.69	86.93	87.95	87.27
Analyzed composition, DM basis				
CP, %	9.3	17.2	21.4	22.5
EE, %	2.9	2.8	4.0	2.3
NDF, %	12.8	16.7	21.8	18.7
ADF, %	3.0	4.6	7.9	7.3
Ash, %	3.4	4.4	5.2	5.3
GE, MJ/kg	18.12	18.23	18.73	18.36
Calculated composition				
SID Lys	0.38	0.80	0.99	0.98
SID Thr/SID Lys	0.70	0.65	0.65	0.67
SID Trp/SID Lys	0.22	0.20	0.21	0.22
SID Val/SID Lys	0.78	0.72	0.74	0.76
SID Leu/SID Lys	2.21	1.55	1.41	1.43
SID Ile/SID Lys	0.50	0.60	0.61	0.61
SID His/SID Lys	0.45	0.44	0.47	0.47
SID Met + Cys/SID lys	0.98	0.59	0.63	0.64
SID Phe + Tyr/SID Lys	1.34	1.29	1.20	1.17

^a^ All samples were analyzed in duplicate, SBM, soybean meal; EP-RSM, expelled press rapeseed meal; SE-RSM, solvent extracted rapeseed meal

^b^Vitamin-mineral premix supplied the following per kg of diet: vitamin A, 5,512 IU; vitamin D_3_, 2,200 IU; vitamin E, 30 IU; vitamin K_3_, 2.2 mg; vitamin B_12_, 27.6 μg; riboflavin, 4 mg; pantothenic acid, 14 mg; niacin, 30 mg; choline chloride, 400 mg; folic acid, 0.7 mg; thiamine, 1.5 mg; pyridoxine, 3 mg; biotin, 44 μg; Mn (MnO), 40 mg; Fe (FeSO_4_ · H_2_O), 75 mg; Zn (ZnO), 75 mg; Cu (CuSO_4_ · 5H_2_O), 100 mg; I (KI), 0.3 mg; Se (Na_2_SeO_3_), 0.3 mg

During each period, pigs were individually housed in metabolism crates for 16 d, which included seven initial days to adapt to the feed, metabolism crate and environmental conditions. On d 8, the pigs were transferred to the open-circuit respiration chambers [9, 10] for measurement of daily O_2_ consumption and CO_2_ and CH_4_ productions. During this time, pigs were fed one of the four diets at 2,200 kJ ME/(kg BW^0.6^·d). Total feces and urine were collected from d 9 to d 13; gas exchanges were measured over the same period for calculating heat production (HP). On d 14 and 15, pigs were fed at their maintenance requirement level (890 kJ ME/(kg BW^0.6^·d) [9] in order to adapt them from the fed to the fasted state. HP was also measured at this low feed level, but the results are not included in the present paper. On the last day of each period (d 16), pigs were fasted; the HP measured during the last 8 h of d 16 from 2230 h (d 16) to 0630 h (d 17) was considered as fasting heat production (FHP). The FHP period started then 31 h after the last meal and always in the dark with expected minimal physical activity.

Pigs were fed equal size meals twice daily at 15÷30 and 1530 h and had free access to water via a low-pressure nipple drinker throughout the trial. The chambers were opened for approximately 1 h per day at 0830 and 1530 h to feed the pigs and collect the feces. The O_2_ consumption and CO_2_ and CH_4_ productions during this time were not included in the calculation of daily HP. The concentration of CO_2_ in the chamber increased when the door was closed. The calculation of HP began when the concentration of CO_2_ in the chamber was above 2,000 ppm [10].

During d 9 to d 13, feed refusals and spillage were collected twice daily and dried and weighed. Total but separate collections of feces and urine were conducted according to the methods described by Li et al. [10]. Feces were collected twice daily at 0830 and 1530 h when the chamber door was opened and immediately stored at −20 °C. Urine was collected each morning at 0830 h for each pig from plastic buckets containing 50 mL of 6 N HCl and filtered through cotton gauze. The total urinary volume produced by each pig was measured and 5% of the daily urinary excretion was stored at −20 °C. At the end of the urinary collection, urine samples were thawed, and thoroughly mixed, and a sub-sample was saved for analysis. Urine was collected separately during the 24 h fasting state to calculate urinary nitrogen (N) losses for the calculation of FHP.

At the end of the experiment, fecal samples were thawed, mixed, weighed, and sub-samples were oven-dried for 72 h at 65 °C for measurement of dry matter fecal excretion. The feed and fecal samples were ground through a 1-mm screen prior to chemical analysis. Six chambers were available for the current experiment. Therefore, Exp.1 was conducted at four consecutive periods; the experimental design is detailed in the Table 7 in Appendix.

### Exp.2: Growth performance experiment

A total of 175 growing pigs (initial BW = 36.0 ± 5.2 kg) were fed 1 of 5 diets for 28 d, with five pigs per pen and seven replications (pens) per treatment. Diets were a corn-soybean meal based diet and diets including 10% or 20% EP-RSM and 10 or 20% SE-RSM (Table 3). All ingredients in Exp. 2 (corn, SBM, EP-RSM and SE-RSM) were the same as in Exp. 1; the NE values of diets in Exp. 2 were calculated according to the results of Exp. 1 and the ratio between SID Lys and NE value was the same among the five diets. The ratio between SID essential amino acids and SID Lys met the ideal protein profile. Pigs had free access to feed and water throughout the experiment; room temperature was maintained between 22 and 24 °C.

**Table 3:** Ingredients and chemical composition of the Exp.2 diets^a^

Diets	Basal	EP-RSM		SE-RSM	
		10%	20%	10%	20%
Ingredients, %					
Corn	76.64	69.24	61.82	69.35	62.01
Soybean meal	20.00	18.00	16.00	18.00	16.00
EP-RSM	-	10.00	20.00	-	-
SE-RSM	-	-	-	10.00	20.00
Dicalcium phosphate	0.60	0.40	0.35	0.45	0.35
Limestone	0.75	0.75	0.60	0.70	0.60
Salt	0.35	0.35	0.35	0.35	0.35
Vitamin and mineral premix^b^	0.50	0.50	0.50	0.50	0.50
Lys-HCl	0.50	0.36	0.22	0.33	0.16
Met	0.20	0.12	0.05	0.10	0.03
Thr	0.18	0.11	0.05	0.09	0.00
Trp	0.07	0.04	0.01	0.03	0.00
Val	0.21	0.13	0.05	0.10	0.00
Calculated composition					
CP	15.67	17.51	19.35	17.60	19.57
Ca	0.53	0.53	0.52	0.53	0.52
STTD P	0.26	0.24	0.26	0.25	0.25
DE^c^	14.25	14.28	14.32	13.92	13.59
ME^c^	13.75	13.76	13.77	13.37	12.99
NE^c^	10.49	10.48	10.47	10.20	9.92
SID Lys^d^	1.03	1.03	1.03	1.00	0.98
SID Lys/NE, g/MJ	0.98	0.98	0.98	0.98	0.98
SID Thr/SID Lys	0.65	0.65	0.65	0.65	0.65
SID Trp/SID Lys	0.19	0.19	0.19	0.19	0.19
SID Val/SID Lys	0.76	0.76	0.76	0.76	0.76
SID Leu/SID Lys	1.20	1.28	1.35	1.32	1.44
SID Ile/SID Lys	0.50	0.53	0.58	0.54	0.62
SID His/SID Lys	0.35	0.40	0.45	0.41	0.47
SID Met + Cys/SID lys	0.55	0.55	0.57	0.55	0.58
SID Phe + Tyr/SID Lys	1.00	1.08	1.15	1.09	1.18

^a^A total of 175 pigs were used in an 28-d study. There were five pigs per pen and seven pens per treatment; SBM, soybean meal; EP-RSM, expelled press rapeseed meal; SE-RSM, solvent extracted rapeseed meal

^b^Vitamin-mineral premix supplied the following per kg of diet: vitamin A, 5,512 IU; vitamin D_3_, 2,200 IU; vitamin E, 30 IU; vitamin K_3_, 2.2 mg; vitamin B_12_, 27.6 μg; riboflavin, 4 mg; pantothenic acid, 14 mg; niacin, 30 mg; choline chloride, 400 mg; folic acid, 0.7 mg; thiamine, 1.5 mg; pyridoxine, 3 mg; biotin, 44 μg; Mn (MnO), 40 mg; Fe (FeSO_4_ · H_2_O), 75 mg; Zn (ZnO), 75 mg; Cu (CuSO_4_ · 5H_2_O), 100 mg; I (KI), 0.3 mg; Se (Na_2_SeO_3_), 0.3 mg

^c^DE, ME and NE calculated from values measured in Exp. 1

^d^SID values were referenced from NRC (2012)

### Chemical analyses and calculations

Ingredients, diets and feces were analyzed for dry matter (DM) (AOAC 2007, 930.15), crude protein (CP) (AOAC 2007, 984.13), ash (AOAC 2007, 942.05) [11], and ether extract (EE) [12]. The organic matter (OM) was calculated as DM minus ash content. Neutral detergent fiber (NDF) and acid detergent fiber (ADF) were determined using filter bags and fiber analyzer equipment (Fiber Analyzer, Ankom Technology, Macedon, NY) following a modification of the procedure of Van Soest et al. [13]. The gross energy (GE) was analyzed using an isoperibol calorimeter (Parr 6300 Calorimeter, Moline, IL) with benzoic acid as a standard.

The ingredients were hydrolyzed with 6 N HCl at 110 °C for 24 h and analyzed for 15 amino acids using an Amino Acid Analyzer (Hitachi L-8900, Tokyo, Japan). Methionine and cysteine were determined as methionine sulfone and cysteic acid after cold performic acid oxidation overnight and hydrolyzing with 7.5 N HCl at 110 °C for 24 h using an Amino Acid Analyzer (Hitachi L-8800, Tokyo, Japan). Tryptophan was determined after LiOH hydrolysis for 22 h at 110 °C using HPLC (Agilent 1200 Series, Santa Clara, CA, USA).

In Exp.1, the DM intake from d 9 to d 13 in each period was calculated as the product of feed intake and DM content of diets. GE intake was calculated as the product of the GE content of the diet and the actual feed DM intake over the 5-d collection period from d 9 to 13. The energy lost in feces, urine and methane were measured for each animal on a given diet. The ME included energy lost as urine and methane. Energy lost as methane was calculated using a 39.54 kJ/L conversion factor [14].

During d 9 to d 13 of each period, O_2_, CO_2_, and CH_4_ concentrations in both ingoing and outgoing air, and outgoing air flow rates were measured at 5 min intervals. These concentrations were then used to calculate O_2_ consumption and CO_2_ and CH_4_ productions during each 5 min interval and these values were averaged and extrapolated to a 24 h period. Total heat production (THP) was then calculated for each day from gas exchanges and urinary loss of N according to Brouwer [14] using the following equation:

$$ \mathrm{T}\mathrm{H}\mathrm{P}\left(\mathrm{kJ}\right)=16.18\times {\mathrm{O}}_2\left(\mathrm{L}\right)+5.02\times {\mathrm{CO}}_2\left(\mathrm{L}\right)\hbox{-} 2.17\times {\mathrm{CH}}_4\left(\mathrm{L}\right)\hbox{-} 5.99\times \mathrm{urinaryN}\left(\mathrm{g}\right) $$

Retention of energy (RE) was calculated according to the following equation:

$$ \mathrm{R}\mathrm{E}\left(\mathrm{MJ}/\mathrm{d}\right)=\left[\mathrm{MEintake}\left(\mathrm{MJ}/\mathrm{d}\right){\textstyle \hbox{-}}\mathrm{THP}\left(\mathrm{MJ}/\mathrm{d}\right)\right] $$

Retention of energy as protein (RE_P_) was calculated from nitrogen balance and the retention of energy as lipid (RE_L_) corresponded to the difference between RE and RE_P_ [10].

FHP was calculated using the equation used for THP with gas concentrations and air flow obtained from only the last 8-h heat production measurement on d 16 (ie, from 2230 to 0630 h or started 31 h after the last maintenance meal) [9]. In order to base production using the same time span as used for THP, the 8-h heat production was extrapolated to a 24-h period. Net energy of each diet was calculated according to Noblet et al. [15]

$$ \mathrm{N}\mathrm{E}\left(\mathrm{kJ}/\mathrm{kg}\ \mathrm{DM}\right)=\left[\mathrm{RE}\left(\mathrm{kJ}/\mathrm{d}\right)+\mathrm{FHP}\left(\mathrm{kJ}/\mathrm{d}\right)\right]/\mathrm{D}\mathrm{M}\ \mathrm{intake}\left(\mathrm{kg}/\mathrm{d}\right) $$

The FHP value obtained on the fasting day [kJ/(kg BW^0.6^·d)] was used during the fed days according to the estimated average BW over the fed days.

The DM of ingredients was measured prior to the preparation of diets in order to calculate the DM ratio of each test ingredient in the diet. The DE, ME and NE of the corn was calculated using direct method. The percentage minerals and vitamins in the corn diet was 2.79% (DM basis) and was not a source of energy; therefore, the energy values of the corn and amino acids were divided by 0.9721; the energy of corn per se was calculated by subtracting the energy of amino acids (Evapig). The difference method was used to calculate the average GE, DE, ME and NE contributions of soybean meal assuming that the average GE, DE, ME and NE values of the corn and amino acids mixture estimated from the corn diet were applicable to the corn-soybean meal diet [16]. The DE/GE, ME/DE and NE/ME ratios could then be calculated for soybean meal from these calculated GE, DE, ME and NE values and used to estimate the final DE, ME and NE values as the product of measured GE and DE/GE for DE, measured GE and DE/GE and ME/DE for ME and measured GE and DE/GE and ME/DE and NE/ME for NE. All calculations were done on a DM basis. Similarly, the corn-soybean meal diet was used as the basal diet when the energy of rapeseed meal was calculated according to the difference method. The apparent total tract digestibility (ATTD) of nutrients in diets was calculated according to the methods of Noblet et al. [15]. The respiratory quotient (RQ) corresponds to the ratio between CO_2_ production and O_2_ consumption.

In Exp. 2, all pigs were weighed on d 1and d 29 to determine average daily gain (ADG), average daily feed intake (ADFI) and gain to feed ratio (G:F). The caloric efficiency of dietary energy was calculated as ADFI (kg/d) × Diet energy content (MJ/kg)/ADG (kg/d) with energy content expressed as DE, ME or NE.

### Statistical analysis

Data from Exp. 1 were analyzed using PROC MIXED (SAS Inst. Inc., Cary, NC, USA) with diet as fixed effects and period and chamber as random effects. When the F-test for diet treatment was significant, differences between dietary treatments were tested among least square means using Tukey’s least significant difference. Data in Exp. 2 were analyzed using MIXED procedure of SAS for a completely randomized design with fixed effects of treatment and pen as the experiment unit. In all analyses, the differences were considered significant if *P* < 0.05, and considered as a trend at *P <* 0.10.

## Results

### Chemical composition of rapeseed meal

The percentage of EE in EP-RSM was greater than in SE-RSM (9.5% vs. 1.1%), while the CP content in the EP-RSM was lower than in the SE-RSM (39.2% vs. 42.4%). The NDF content in the EP-RSM was greater than in SE-RSM (37.0% vs. 30.8%). These two rapeseed meals contained similar proportions of amino acids. The composition of the four diets is in agreement with the ingredients percentages and their composition with the lowest CP content in the corn diet and the highest CP contents in the rapeseed meal diets. Finally, the EE content was higher in the EP-RSM diet.

### Exp.1: Net energy experiment

The ATTD of DM, OM and GE was similar in the corn and corn-soybean meal diets, which values were greater than in the two rapeseed meal diets (Table 4, 90.2% vs. 86.6% for ATTD of DM, 90.3% vs. 87.0% for ATTD of OM, 88.3% vs. 85.1% for ATTD of GE; *P* < 0.01). However, the ATTD of CP was similar in the corn-soybean meal diet and the two rapeseed meal diets, these values being greater than in the corn diet in connection with its lower CP content (86.4% vs. 79.9%; *P* < 0.01).

**Table 4:** Effect of diet characteristics on energy and nitrogen balances of growing pigs (Exp. 1)^1^

Item	Corn	SBM	EP-RSM	SE-RSM	SEM	*P* - value
BW, kg	40.2^b^	44.0^a^	44.1^a^	44.1^a^	0.9	0.01
DM intake, kg/d	1.33	1.34	1.37	1.40	0.02	0.07
Digestibility coefficients, %						
DM	90.4^a^	90.0^a^	87.3^b^	85.8^b^	0.7	<0.01
CP	79.9^b^	88.0^a^	86.2^a^	85.1^a^	1.3	<0.01
OM	90.4^a^	90.2^a^	87.7^b^	86.3^b^	0.7	<0.01
GE	88.2^a^	88.3^a^	86.2^ab^	84.0^b^	0.8	<0.01
Nitrogen balance, g/d						
Intake	19.9^d^	37.0^c^	46.9^b^	50.4^a^	0.6	<0.01
Fecal output	4.0^b^	4.5^b^	6.6^a^	7.5^a^	0.5	<0.01
Urinary output	6.6^b^	10.6^ab^	15.5^a^	15.4^a^	1.4	<0.01
Retention	9.4^b^	21.9^a^	24.8^a^	27.5^a^	1.2	<0.01
Energy utilization, %						
Urinary energy % of DE	1.8^c^	2.7^b^	3.4^ab^	3.9^a^	0.25	<0.01
Methane energy, % DE	0.4	0.7	0.4	0.6	0.08	0.06
ME/DE	97.8^a^	96.6^b^	96.2^bc^	95.5^c^	0.27	<0.01
NE/ME	78.2	76.5	76.3	76.7	0.82	0.16
Energy balance, kJ/(kg BW^0.6^·d)						
ME intake,	2,273^a^	2,160^bc^	2,195^b^	2,120^c^	28	<0.01
THP	1,259	1,308	1,268	1,250	25	0.15
THPc^2^	1,232	1,317	1,265	1,271	22	0.08
RE_P_^3^	152^c^	337^b^	381^ab^	424^a^	20	<0.01
RE_L_^4^	862^a^	516^b^	546^b^	446^b^	34	<0.01
RE	1,014^a^	852^b^	928^b^	870^b^	27	<0.01
FHP	769	809	756	766	22	0.18
RQ						
Fed state	1.11^a^	1.05^b^	1.02^b^	1.03^b^	0.01	<0.01
Fasted state	0.77	0.77	0.78	0.78	0.01	0.07
Energy values, MJ/kg DM						
DE	15.98^a^	16.10^a^	16.14^a^	15.42^b^	0.14	<0.01
ME	15.64^a^	15.56^a^	15.52^a^	14.73^b^	0.13	<0.01
NE	12.23^a^	11.90^a^	11.84^ab^	11.30^b^	0.15	<0.01

^1^A total 24 pigs were allotted to 1 of 4 dietary treatments with six pigs per diet and four periods, 16 d per period; SBM, soybean meal; EP-RSM, expelled press rapeseed meal; SE-RSM, solvent extracted rapeseed meal

^2^THPc means that total heat production was adjusted for comparable ME intake by covariance

^3^RE_P_ = Energy retention as protein [kJ/(kg BW^0.6^·d)] = [N intake (g) – N in feces (g) – N in urine (g)] × 6.25 × 23.86 (kJ/g)/BW^0.6^

^4^ RE_L_ = Energy retention as fat [kJ/kg BW^0.6^·d)]  = [RE (kJ) – energy retention as protein (kJ)]/BW^0.6^

^abcd^Means in the same row with differing superscripts differ (*P* < 0.05)

The nitrogen intake increased with the CP content in the diet (*P* < 0.01). The nitrogen output from feces and urine was similar in the corn-soybean meal diet and the corn diet but lower than in the two rapeseed meal diets (*P* < 0.01). Consequently, the urinary energy as a percentage of DE was greater in the SE-RSM diet than in the corn-soybean meal diet, and the lowest in the corn diet (*P* < 0.01). These led to ME to DE ratio greater in the corn diet than in the corn-soybean meal diet, and the lowest in the SE-RSM diet (*P* < 0.01).

The retention of nitrogen was similar in the two rapeseed meal diets and the corn-soybean diet, which was markedly greater than in the corn diet (24.7 vs. 9.4 g per day, *P* < 0.01). Methane energy averaged 0.54% of DE and it tended to be greater in the SE-RSM diet than in the EP-RSM diet (*P* = 0.07).

Compared to the pigs fed the rapeseed meal diet, the pigs fed the corn-soybean meal diet had a comparable ME intake while the pigs fed the corn diet had the greatest ME intake. There was little difference in THP (average: 1,270 kJ/(kg BW^0.6^·d)) between diets despite differences in ME intake. However, it tended to be lower in the corn diet when THP was adjusted for similar ME intake (Table 4). Consequently, the pigs fed the corn diet had the greatest RE compared to other diets (1,014 vs. 883 kJ/(kg BW^0.6^·d), *P* < 0.01). In connection with their lower nitrogen gain and their higher RE, the greatest RE_L_ was found in the pigs fed the corn diet. The mean FHP was 775 kJ/(kg BW^0.6^·d) and was not affected by diet composition (*P* = 0.19). The RQ was the greatest in the corn diet (*P* < 0.01) in connection with the higher RE_L_. There was no difference in NE to ME ratio among the four diets (*P* = 0.18) despite difference in chemical composition. Lower energy values (DE, ME and NE) were observed in the SE-RSM diets compared to the other diets that had comparable energy values.

The ingredients values calculated from the diets values are shown in Table 5. The ATTD of CP, OM and GE of the four ingredients were quite variable with the lowest value for SE-RSM. The ATTD of OM of the corn was the greatest. Compared to other ingredients, the corn had the greatest ME to DE ratio (97.8%) and NE to ME ratio (78.4%), while the SE-RSM had the least ME to DE ratio (90.7%); the soybean meal had the lowest NE to ME ratio (70.2%). The NE of corn, soybean meal, EP-RSM and SE-RSM diets were 12.46, 11.34, 11.71 and 8.83 MJ/kg DM, respectively.

**Table 5:** Energy utilization and energy value of the four ingredients (Exp. 1)^a^

Item	Corn	SBM	EP-RSM	SE-RSM
Digestibility coefficients, %				
CP	82.4	94.8	81.6	78.9
OM	93.1	90.5	76.4	68.0
GE	88.2	89.8	77.6	65.6
Energy utilization, %				
ME/DE	97.8	92.1	94.9	90.7
NE/ME	78.3	70.2	74.7	76.5
NE/DE	76.6	64.6	71.0	69.4
Energy values, MJ/kg DM				
DE	16.27	17.51	16.55	12.82
ME	15.92	16.10	15.71	11.61
NE	12.46	11.34	11.71	8.83

^a^ There were six pigs per treatment; SBM, soybean meal; EP-RSM, expelled press rapeseed meal; SE-RSM, solvent extracted rapeseed meal

### Exp.2: Growth performance experiment

During the 28 days period, the NE intake averaged 21.15 MJ/d and was similar for the five diets. Similarly, final BW, ADG, ADFI and G:F did not change with the inclusion of EP-RSM or SE-RSM in the diets (Table 6). The mean caloric efficiencies of DE, ME and NE for BW gain were 31.5, 30.3 and 23.1 MJ/kg, respectively and were not affected by diet characteristics (*P* > 0.10).

**Table 6:** Effect of rapeseed meal on growing pigs performance (Exp.2)^a^

Item	Basal	EP-RSM^b^		SE-RSM^c^		SEM	*P*-value
		10%	20%	10%	20%		
Initial BW, kg	35.9	36.0	36.1	36.1	36.1	2.0	0.99
Final BW, kg	62.0	62.2	61.3	61.0	61.5	2.4	0.99
ADFI, g/d	2,093	2,059	1,996	2,019	2,086	75	0.86
ADG, g/d	929	937	901	891	910	24	0.64
G:F	0.446	0.458	0.454	0.442	0.436	0.010	0.71
NE intake, MJ/d	21.96	21.58	20.91	20.60	20.69	0.78	0.68
Caloric efficiency, MJ/kg							
DE	31.93	31.24	31.60	31.47	31.16	0.72	0.95
ME	30.80	30.10	30.39	30.21	29.77	0.70	0.88
NE	23.60	23.00	23.16	23.13	22.78	0.53	0.86

^a^ A total of 175 pigs were used in an 28-d study. There were five pigs per pen and seven pens per treatment

^b^EP-RSM, expelled press rapeseed meal

^c^SE-RSM, solvent extracted rapeseed meal

## Discussion

In the current experiment, the two rapeseed meals are not from the same batch. Therefore, we can’t evaluate the oil extraction processing and its impact on the composition of the non-fat fraction of the meal. However, the lower CP in the high fat RSM is consistent with the dilution effect of residual oil. Overall, the chemical composition and amino acids contents of the four ingredients are within the range of values reported in our previous work [2, 3, 17, 18]. However, the NDF content (33.9% DM basis) in the two rapeseed meals was greater than the values published by Sauvant et al. (31.9% DM basis) [19] and NRC (25.2% DM basis) [20].

As in other literature studies [21, 22], the digestibility of DM, OM and GE were lower in the rapeseed meal diets in connection with their higher NDF content. But this negative effect of dietary fiber from RSM is attenuated in the EP-RSM diet in connection with its high and digestible oil fraction. This resulted in similar digestibility of GE between the EP-RSM and corn-soybean meal diets. These results are also found in the four ingredients with the lowest digestibility DM, OM and GE in the SE-RSM in connection with its high NDF content.

The ME to DE ratio was lower in the rapeseed diets in connection with their high urinary nitrogen output due to the high CP in the rapeseed diets, which also confirmed the result that CP is negatively correlated with ME to DE ratio [15]. The methane energy measured in our study is consistent with data obtained in similar body weight pigs [9, 10, 15] and indicates that the methane energy loss in growing pigs is little variable with differences in diets composition.

The THP values measured in the current experiment are in agreement with the range of THP observed at comparable ME intakes for 61 diets by Noblet et al. [15]. In the current experiment, in order to eliminate the effect of ME intake, the THP was adjusted for comparable ME intakes by covariance. In this way, the THP for the corn diet became lower in connection with the high starch content of the diet and also the higher proportion of energy retained as fat, which is fully consistent with the rather high efficiency of starch for NE [15] and the higher efficiency for fat gain than for protein gain [23]. In the current experiment, the value of FHP (average: 775 kJ/(kg BW^0.6^·d)) was estimated as the nocturnal heat production after a period of feed deprivation of 31 h. The value is remarkably close to our previous work using the same method [10, 24, 25] and the results of Noblet et al. [15]. In addition, in agreement with other literature data [10, 26, 27], the FHP was not affected by diet composition (dietary CP or EE).

In the current Exp.1, the corn diet was supplemented with essential AA to meet the optimal amino acids balance, however, the daily supply was insufficient and the nitrogen gain and RE_P_ are lower. In addition, RE was higher than in the other diets and RE_L_ was then higher in the corn diet. However, we supplemented corn with free AA in order to attenuate this effect of shortage of AA and CP. In this way, the energy value (ME and NE) of corn measured in the Exp.1 could be representative of normal situations where corn is used with optimal levels of essential AA (Exp.2).

The soybean meal had the lowest NE to ME ratio, while the corn had the greatest NE to ME ratio. This indicates that the efficiency of utilization of ME for NE depends on chemical composition [27]. The NE to ME ratio of corn is also similar to the value in Sauvant et al. (78.3% vs. 80.1%) [28]. However, the NE to ME ratio of soybean meal and rapeseed meal obtained in the present trial are greater (70.2% vs. 60.5% for soybean meal; 76.5% vs. 59.7% for rapeseed meal) than the values in Sauvant et al. [28]. The difference in NE to ME ratio may be explained by the slightly greater FHP measured in the current experiment than value used by Sauvant et al. [28] (775 vs. 750 kJ/(kg BW^0.6^·d)).

The DE and ME values of the four ingredients measured in the current experiment are similar to those obtained in our previous studies [2, 3, 17, 18]. In agreement with the values of NE to ME ratio in the present study and in Sauvant et al. [28], the NE value of corn measured in Exp. 1 is similar to the value in Sauvant et al. [28]. But the NE values of soybean meal and rapeseed meal are greater than the table values in Sauvant et al. (11.34 vs. 9.22 MJ/kg DM for SBM, 8.83 vs. 7.14 MJ/kg DM for SE-RSM) [28]. The NE contents in corn, soybean meal were similar to the values reported by Liu et al. (12.46 vs. 13.21 MJ/kg DM for corn, 11.34 vs. 10.62 MJ/kg DM for SBM) [24] using the same methodology. The NE content in SE-RSM measured using indirect calorimetry by Heo et al. (8.83 vs. 8.80 MJ/kg DM) [29] was similar to the value in the present experiment. The greater NE value found in EP-RSM than in SE-RSM is associated with its high EE value, which was in agreement with the results by Woyengo et al. [30].

In the growth trial, diets were supplemented with crystalline amino acids in order to maintain a constant SID Lys/NE ratio (0.98 g/MJ) and balanced for the other essential amino acids. Therefore, the efficiencies of energy for BW gain were calculated without the confounding effect of amino acids deficiency. Several research projects reported that there was no impairment of pig performance when EP-RSM or SE-RSM replaced soybean meal in growing pig diets formulated to equal NE and SID amino acids [31, 32], or that contained equal quantities of digestible CP and digestible amino acids [4]. In the current experiment, we also failed to detect any statistically significant differences in growth performance with increasing inclusion rates of EP-RSM or SE-RSM.

In the current research, we used the same ingredients (corn, soybean meal, EP-RSM and SE-RSM) in the two experiments. Therefore, the NE of the five diets in Exp.2 was calculated according to the values measured in Exp. 1. Increasing inclusion level of EP-RSM or SE-RSM did not affect caloric efficiency of NE, which indicated that the NE value measured in Exp.1 by indirect calorimetry was close to the actual value as indicated by the caloric efficiency results in Exp. 2. In the growth trial (Exp. 2), we also failed to detect any statistically significant differences in caloric efficiency of DE and ME with increasing inclusion rates of EP-RSM or SE-RSM. Therefore, these data do not show any advantage of NE system on DE or ME systems. In fact, the diets in the growth trial were very similar in terms of chemical composition, energy value, CP and AA contents with then little expected differences in ME/DE and NE/ME ratios. Furthermore, our research to measure the caloric efficiency of NE was only according to ADG. This should also be adjusted for differences in BW gain composition (lipid or fat contents) for being more accurate.

## Conclusions

The DE, ME and NE values in EP-RSM were greater than values in SE-RSM. The efficiency of utilization of ME for NE depends on chemical composition. The NE values measured in Exp.1 are confirmed by results of Exp. 2 with comparable caloric efficiencies of NE for all diets.

## Appendix

**Table 7:** Design of net energy experiment (Exp.1)

	Chambers	A1	A2	B1	B2	C1	C2
Period 1	Diets	I	II	III	IV	I	II
	Pigs	1	2	3	4	5	6
Period 2	Diets	II	III	IV	I	II	III
	Pigs	7	8	9	10	11	12
Period 3	Diets	III	IV	I	II	III	IV
	Pigs	13	14	15	16	17	18
Period 4	Diets	IV	I	II	III	IV	I
	Pigs	19	20	21	22	23	24

## Abbreviations

AA: Amino acid
ADF: Acid detergent fiber
ADFI: Average daily feed intake
ADG: Average daily gain
ATTD: Apparent total tract digestibility
BW: Body weight
CP: Crude protein
DE: Digestible energy
DM: Dry matter
EE: Ether extract
EP-RSM: Expeller-pressed rapeseed meal
F:G: Gain to feed ratio
FHP: Fasting heat production
GE: Gross energy
HP: Heat production
ME: Metabolizable energy
N: Nitrogen
NDF: Neutral detergent fiber
NE: Net energy
OM: Organic matter
RE: Retention energy
RE_L_: Retention energy as lipid
RE_P_: Retention energy as protein
RQ: Respiratory quotient
SEM: Standard error of the mean
SE-RSM: Solvent-extracted rapeseed meal
SID: Standard ileal digestibility
THP: Total heat production
